# Cacao biotechnology: current status and future prospects

**DOI:** 10.1111/pbi.12848

**Published:** 2017-11-19

**Authors:** Anushka M. Wickramasuriya, Jim M. Dunwell

**Affiliations:** ^1^ Department of Plant Sciences Faculty of Science University of Colombo Colombo Sri Lanka; ^2^ School of Agriculture, Policy and Development University of Reading Reading UK

**Keywords:** *Theobroma cacao*, chocolate, somatic embryogenesis, genetics, genomics, breeding, transformation

## Abstract

*Theobroma cacao*—The Food of the Gods, provides the raw material for the multibillion dollar chocolate industry and is also the main source of income for about 6 million smallholders around the world. Additionally, cocoa beans have a number of other nonfood uses in the pharmaceutical and cosmetic industries. Specifically, the potential health benefits of cocoa have received increasing attention as it is rich in polyphenols, particularly flavonoids. At present, the demand for cocoa and cocoa‐based products in Asia is growing particularly rapidly and chocolate manufacturers are increasing investment in this region. However, in many Asian countries, cocoa production is hampered due to many reasons including technological, political and socio‐economic issues. This review provides an overview of the present status of global cocoa production and recent advances in biotechnological applications for cacao improvement, with special emphasis on genetics/genomics, *in vitro* embryogenesis and genetic transformation. In addition, in order to obtain an insight into the latest innovations in the commercial sector, a survey was conducted on granted patents relating to *T. cacao* biotechnology.

## Introduction

**Figure 1:**
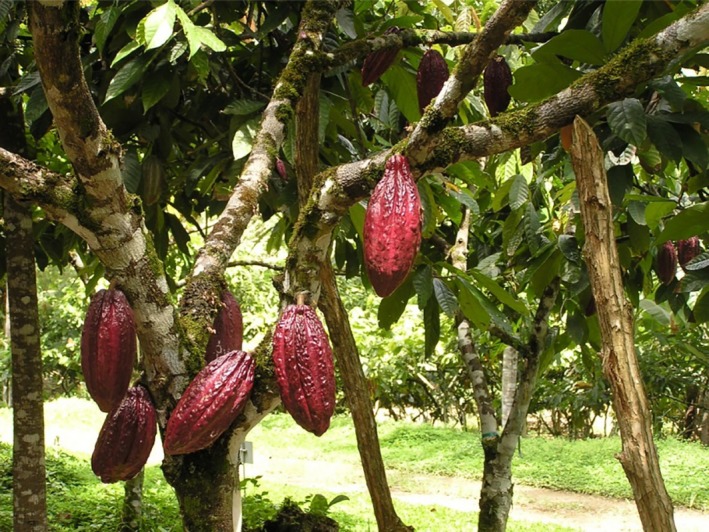
Cacao tree with multiple pods.

The diploid tropical fruit crop species (2*n* = 2*x* = 20), *Theobroma cacao* (cacao) (Figure 1), is an economically important agricultural commodity for millions of people worldwide. It is grown by about 6 million farmers globally, and livelihoods of more than 40 million people depend on cocoa (Beg *et al*., 2017; World Cocoa Foundation, 2012). The majority of world cocoa production (approximately 80%–90%) comes from smallholder farmers (World Cocoa Foundation, 2014). This crop originated from the Amazonian basin (Motamayor *et al*., 2002; Wood and Lass, 1985), and today, it is cultivated in many regions of the humid tropics.

**Figure 2:**
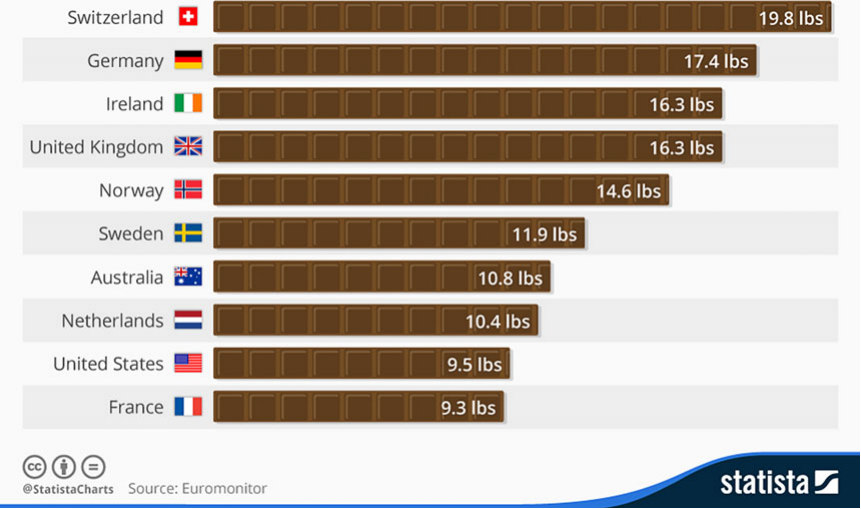
Annual consumption of chocolate per year (modified from Statista.org).

The cocoa beans are the primary source of raw material for the multibillion dollar industry that produces chocolate and associated confectionery products, with Switzerland being the country with the highest consumption (Figure 2), although much of this is due to purchases by tourists to that country. The economic significance of the chocolate industry has been recently reviewed (Squicciarini and Swinnen, 2016), with the global market for chocolate rising 13% from 2010 to reach US$101 billion in 2015.

This crop belongs to the Malvaceae family, and more than 20 species are known within the *Theobroma* genus (Wood and Lass, 1985). Among these, *T. cacao* is the only species that is cultivated extensively (Wood and Lass, 1985). This species has three genetic groups based on morphological and anatomical characteristics—Criollo (*T. cacao* Spp. Criollo), Forastero (*T. cacao* Spp. Sphaerocarpum) and Trinitario (Pridmore *et al*., 2000). Of these, the Criollo type is well known for its superior flavour and provides the raw material from which fine flavour chocolates are produced; these represent 5%–10% of world chocolate production (Rusconi and Conti, 2010). However, increased susceptibility to pest and diseases, low vigour and yield has made this variety less popular among cacao growers. Today, most of the world's chocolate production (approximately 80%) comes from the Forastero type of cacao; this variety is favoured over the Criollo for its disease‐resistant and high‐yielding nature, and beans from this variety are relatively cheaper than those from the Criollo type (Rusconi and Conti, 2010). The third genetic group, Trinitario, is a hybrid produced from crosses between Criollo and Forastero varieties. This variety was initially developed in Trinidad, and today, it is cultivated in many parts of South and Central America, Africa, South‐East Asia and Oceania for its aroma, productivity and disease‐resistant character.

### World cocoa production

**Figure 3:**
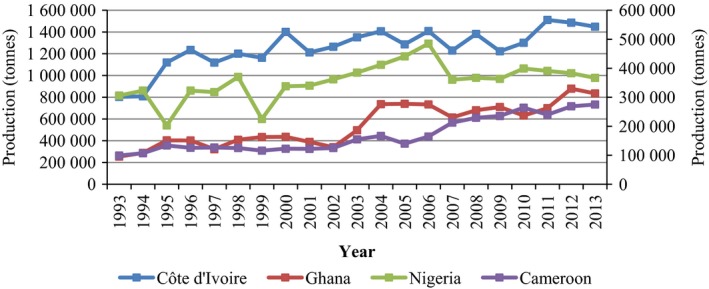
Cocoa bean production in Africa—Côte d'Ivoire, Ghana, Nigeria and Cameroon from 1993 to 2013. Primary axis left: Côte d'Ivoire and Ghana; secondary axis right: Nigeria and Cameroon. Source: FAOSTAT (http://faostat3.fao.org/browse/Q/*/E, retrieved on 7 February 2017).

**Figure 4:**
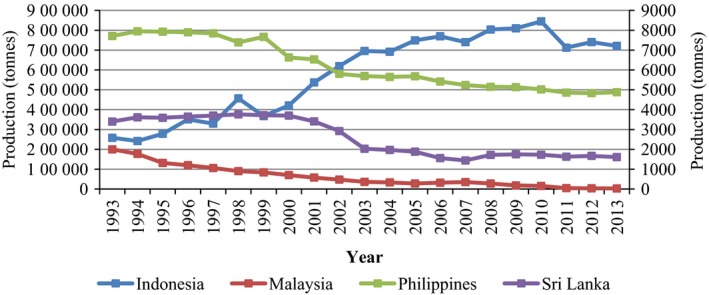
Cocoa bean production in Asia—Indonesia, Malaysia, Philippines and Sri Lanka from 1993 to 2013. Primary axis left: Indonesia; secondary axis right: Malaysia, Philippines and Sri Lanka. Source: FAOSTAT (http://faostat3.fao.org/browse/Q/*/E, retrieved on 7 February 2017).

The International Cocoa Organization (ICCO) estimated that more than 4.0 million metric tons of cocoa beans were produced worldwide in 2015/16 (Pipitone, 2016). Of this total, it is also estimated that Africa contributed approximately 74% (2.92 million tonnes) in the 2015/16 season. This is 5000 tonnes less than the estimated production for 2014/15. Among the cocoa‐producing regions, Côte d'Ivoire, Ghana and Cameroon contributed 1.57, 0.8 and 0.23 million tonnes, respectively, to global production in 2015/16 (Pipitone, 2016). It is important to note that there is a discrepancy between the cocoa bean production data published by the Food and Agriculture Organization of the United Nations (FAO) and the production estimates by the ICCO. This is mainly due to the use of different sources to estimate the production data. Figures 3 and 4 in the present review are based on the data published by the FAO. Figure 3 shows cocoa bean production (tonnes) from 1993 to 2013 in the leading production regions in Africa, namely Côte d'Ivoire, Ghana, Nigeria and Cameroon. With the exception of Cameroon, a slight decline in production from 2012 to 2013 is noted in the other African countries considered.

In addition, the Americas region (16%, 0.64 million tonnes), and Asia and Oceania (10%, 0.4 million tonnes) are ranked as the second and third largest producers of cocoa beans worldwide. At present, Indonesia is the third largest producer after Côte d'Ivoire and Ghana, with an estimated production of 0.33 million tonnes in 2015/16 (Pipitone, 2016). However, production is still relatively low in many Asian countries such as Malaysia, The Philippines and Sri Lanka, which all have a tremendous potential to grow cacao (Figure 4). In addition to the fact that cocoa bean production contributes significantly to the economy of the growing regions, it also serves as a main source of income for millions of smallholder farmers (Darkwah and Verter, 2014).

The demand for cocoa is increasing considerably (approximately 3% per year) (World Cocoa Foundation, 2014). At present, global cocoa production is considered to be at risk, and it has been reported that the world may experience a cocoa shortage by 2020 (Earth Security Group, 2015; Jégourel, 2016). However, the surplus seen in the 2016/17 crop season (up to May 2017) in some cacao growing areas such as Cote d'Ivoire and Ghana is favourable for the future cocoa sector; an 18% increase in the world production is expected for the current crop season as compared to that of the previous season (International Cocoa Organization, 2017).

### Uses of cocoa

This perennial shade grown tree crop provides biodiversity benefits. It is cultivated either as monocultures or in association with other crops such as fruit crops (Guiltinan *et al*., 2008). Cocoa beans are the key raw materials in the production of chocolate and other cocoa‐based products. However, the freshly harvested cocoa beans do not contain the determinants of chocolate aroma or flavour, and hence, postharvesting processing of raw beans (fermentation, drying and roasting) is essential for optimum flavour formation (De Vuyst and Weckx, 2016; Kadow *et al*., 2015; Kongor *et al*., 2016; Loureiro *et al*., 2017). The process of cocoa bean fermentation is trigged by action of micro‐organisms (e.g. yeast, acetic acid bacteria and lactic acid bacteria) (Illeghems *et al*., 2015; de Melo Pereira *et al*., 2016), and the flavour precursors such as organic acids, reducing sugars and free amino acids are produced at the end of the process. In addition, the process of fermentation involves significant reduction in polyphenols (epicatechin and catechin) and alkaloids (methylxanthines caffeine, theobromine) found in raw cocoa beans that give rise to bitterness and unpleasant astringency (Kadow *et al*., 2015; Lee *et al*., 2016).

Most production of cocoa takes place in the tropics, and the beans produced in this region used to be principally processed elsewhere into cocoa powder and cocoa butter (Wood and Lass, 1985). Now, although most of the cocoa grindings (38%) are carried out in the Europe and Russia region (principally the Netherlands), the remainder is processed close to production areas in the Americas (22%), Asia and Oceania (21%) and Africa (19%) (Pipitone, 2016). In addition to its use in confectionery, cocoa products are also considered to have other functional properties (Konar *et al*., 2016; Wilson and Hurst, 2015) and are used in a range of pharmaceutical and cosmetic products. Cacao seeds are a rich source of polyphenolic antioxidants, and consequently, it has been reported that cocoa‐based products contribute a greater proportion of the dietary intake of phenolic antioxidants than do green tea, wine, soya beans and blueberries, which are known antioxidant‐rich food products and beverages (Lee *et al*., 2003). The antioxidant properties of cocoa, particularly the high flavonoid content, are now of great interest due to its profound effects on human health. Specifically, the claim that cocoa polyphenols could prevent cancer or delay/slow down the progression of cancer (chemo‐preventive agents) has received increased attention (Martin *et al*., 2013). Furthermore, flavonoids extracted from cocoa have been shown to play a pivotal role in mediating innate and acquired immunity (Ramiro‐Puig and Castell, 2009), and also have been shown to have an effect on diet induced obesity and insulin resistance (Dorenkott *et al*., 2014). Emerging data support the suggestion that cocoa flavanols may serve as cardioprotective agents. These compounds have been reported to modulate mediators of inflammation (Keen *et al*., 2005). Cocoa flavanols and procyanidins (Bowser *et al*., 2017; Liu *et al*., 2015) have been shown to have beneficial effects including decreased platelet aggregation through increasing concentration of epicatechin and catechin in the plasma (Keen *et al*., 2005; Murphy *et al*., 2003). Furthermore, cacao shell is a rich source of theobromine and vitamin D. The pods contain a high level of potash that is used in soap production (Bart‐Plange and Baryeh, 2003).

### Cocoa bean quality

It is also important to maintain or enhance bean quality. Recently, several bean quality attributes, both physical and chemical, that are required by the cocoa manufacturers/buyers, have been documented in detail to encourage the cacao community towards the production of better quality cocoa (CAOBISCO/ECA/FCC, 2015). These quality characteristics include flavour, purity or wholesomeness (e.g. free from bacteria, infestation, allergens, mycotoxins, heavy metals and pesticide residues), physical characteristics (e.g. consistency, yield of edible material bean, bean size and uniformity, shell content, fat content and moisture content) and cocoa butter characteristics (e.g. free fatty acid content). Some of these bean quality attributes, such as total fat content, acidity, total phenols, organic acids, heavy metals, amino acids, caffeine, theobromine, pH, sugars, macro‐ and micronutrient content, have been considered in the proposed Cocoa Quality Index (CQI) for Forastero‐type beans (Araujo *et al*., 2014). Such an indexing system may represent a useful tool in research programmes designed to improve bean quality for sustainable cocoa production. One recommended source of information on cocoa quality is the Cocoa Atlas (Rohsius *et al*., 2010; http://www.cocoa-atlas.org/). This DVD is funded by the German Cocoa and Chocolate Foundation and produced by the Cocoa Research Group of the Biocenter Klein‐Flottbek, University of Hamburg, Germany. It includes, in addition to global data, valuable information from 32 individual countries, with information from each country divided into 12 sections with the following titles: background notes, cocoa growing areas, cocoa production, cocoa trade, foreign trade, aroma description, bean weight/count, cut test, fat composition, free amino acids, further compounds and pictures of samples. Most recently, a Working Group on the Development of International Standards for the assessment of cocoa quality has been established by the Cocoa of Excellence (CoEx) programme and an initial draft document on this theme is now available (Cocoa of Excellence, 2017).

The flavour profile of beans is a key quality measure in cocoa. For instance, the clone CCN 51, which is planted extensively in Ecuador, exhibits many attractive agronomic traits like disease resistance, high butter content and high productivity; however, it is less popular among fine flavour chocolate manufacturers, especially due to the lack of fine flavour trait (Boza *et al*., 2014). In addition to the cacao genotype, several other factors such as location where the trees are grown (i.e. soil condition), the age of trees and postharvest treatments (fermentation, drying and roasting) also affect cocoa bean flavour (Kongor *et al*., 2016). A comprehensive overview of factors affecting cocoa flavour attributes has been published elsewhere (Afoakwa *et al*., 2008; Kongor *et al*., 2016). Furthermore, a sensory study conducted on raw cacao seeds and fruit pulp using a gas chromatography‐mass spectrometry method has identified monoterpenes, methylketones, and secondary alcohols and their respective esters as the main volatile aroma components in fine flavour clones such as SCA6 and EET62 (Kadow *et al*., 2013). Analytical methods such as MS fingerprinting (Qin *et al*., 2017; Tran *et al*., 2015) and the near‐infrared spectroscopy (NIRS) method (Krähmer *et al*., 2015) have been successfully employed in evaluation of cocoa biochemical quality parameters related to flavour attributes and quality of fermentation, as efficient and routinely applicable approaches. These studies have provided a foundation for understanding the molecular basis of fine aroma components in cocoa, and thereby for the development of molecular markers linked to fine aroma quality in this species.

Good preharvest and postharvest practices are key to maintaining many of the above mentioned bean quality descriptors (CAOBISCO/ECA/FCC, 2015). For instance, selection of suitable planting materials or the desired genetic background for cultivation is necessary to maintain the required flavour, yield, bean size and colour, and cocoa butter content (CAOBISCO/ECA/FCC, 2015; Loureiro *et al*., 2017). Furthermore, the quality of soil in which the cacao plants are grown is also a concern today as there is some evidence for the presence of heavy metals, especially cadmium, in cocoa beans produced in some parts of the producing countries (Arévalo‐Gardini *et al*., 2017; CAOBISCO/ECA/FCC, 2015; Loureiro *et al*., 2017).

Cocoa bean quality is also influenced by postharvest practices, especially the fermentation and drying processes. For example, controlled drying of the fermented cocoa beans is a crucial step to avoid development of off‐flavours that may affect quality of beans. High‐throughput molecular analysis tools could be used for rapid and efficient identification of microbial population diversity during cocoa fermentation and drying, and for development of microbial markers associated with the process (Hamdouche *et al*., 2015). For instance, the powerful biotyping tool, matrix‐assisted laser desorption ionization time‐of‐flight mass spectrometry (MALDI‐TOF MS) method, has recently been used for molecular identification of micro‐organisms involved in cocoa bean fermentation (Miguel *et al*., 2017; Schwenninger *et al*., 2016). Recently, fermentation‐like incubation systems or laboratory‐scale fermentation methods have proven to be applicable for the fermentation process of fresh cacao seeds (Evina *et al*., 2016; Kadow *et al*., 2015). This system, which does not depend on micro‐organisms, may provide a better alternative to the natural fermentation process that is usually difficult to control. Furthermore, the experimental model described in Lee *et al*. (2016) for cocoa fermentation that mimics on‐farm cocoa fermentation process may speed‐up fermentation studies at a laboratory level in the future.

Another important factor that influences the quality of cocoa beans is the specific environmental condition in which cacao plants are cultivated. The increasing atmospheric temperature and evapotranspiration caused by global warming are likely to have a profound impact on global cacao cultivation (Oyekale *et al*., 2009). Additionally, the climate in cacao growing regions has a considerable impact on cocoa fermentation and drying processes. Läderach *et al*. (2013) have projected that by 2050, the present cacao farming areas or cacao‐favoured growing areas in Côte d'Ivoire and Ghana may shift to areas with higher altitudes due to progressive increase in temperatures. A more recent detailed study of this topic is that by Schroth *et al*. (2016). If the predicted climate and weather variability continues, this may have an impact on the economic status of cocoa farmers and major cocoa‐producing countries; as result, global chocolate and confectionery industry is likely to be affected due to a cocoa shortage. Breeding for climate‐smart cacao varieties is vitally important to long‐term sustainability of cocoa production (World Cocoa Foundation, 2016). This subject of climate‐smart agriculture (CSA) is the basis of the ongoing project—“Mainstreaming CSA practices in cocoa production in Ghana,” which aims to implement CSA practices with cacao farmers (http://www.sustainablefoodlab.org/initiatives/climate-smart-agriculture/).

### Cacao genetics and breeding

Cacao is a diploid fruit crop species with a relatively small genome, organized into ten chromosomes (da Silva *et al*., 2017); that is, the genome size is approximately double that of *Arabidopsis thaliana*, the model dicot. Recently, the published genome of the most cultivated type of cacao, *T. cacao* Matina 1‐6 clone reports a genome size of 445 Mbp (Motamayor *et al*., 2013), which is considerably larger than the previously published genome of a Criollo genotype (430 Mbp) (Argout *et al*., 2011). According to the genome statistics reported by Argout *et al*. (2011), 28 798 protein coding genes from more than 682 gene families are present in the cacao genome. These include many genes related to disease resistance, lipid biosynthesis (Zhang *et al*., 2015), flavonoid biosynthetic pathway and terpenoid synthesis (Argout *et al*., 2011). The updated version of this Criollo sequence, with 99% of genes anchored to the 10 chromosomes, was released in January 2017 and is accessible at the Cocoa Genome Hub (http://cocoa-genome-hub.southgreen.fr/) (Argout *et al*., 2017). Availability of whole‐genome sequences for several cacao varieties (Argout *et al*., 2011; Motamayor *et al*., 2013) has allowed identification and characterization of novel genes of interest to breeders and also development of molecular markers for marker‐assisted selection (MAS) (Lopes *et al*., 2011). The release of cacao genome sequences has also provided the way for rapid identification, functional and structural characterization of many gene families in cacao, through *in silico* computational studies and expression analysis. For example, recently three legumain proteins, *Tc*LEG3, *Tc*LEG6 and *Tc*LEG9, which play diverse roles in programmed cell death, seed germination and seed development have been identified and characterized through *in silico* analyses, three‐dimensional modelling and expression analyses (Santana *et al*., 2016). Also, comprehensive genomewide analysis of pathogenesis‐related (PR) gene family in the two published *T. cacao* genomes has identified a set of candidate genes that are likely to be involved in mediating defence responses against major pathogens such as *Phytophthora palmivora* (Maora *et al*., 2017) and *Colletotrichum theobromicola* (Fister *et al*., 2016a). Arrays constructed from subtractive libraries have also been used in an investigation of molecular responses to cocoa black pod infection (Legavre *et al*., 2015). Such findings may contribute to a better understanding of the genetics and genomics of *T. cacao*.

In terms of breeding targets, these can be divided into two main categories. The first is associated with resistance to biotic stress, as unfortunately, outbreaks of diseases (Bailey and Meinhardt, 2016) in major cacao growing areas have significantly affected production in South America and Africa. For instance, Witches’ broom disease (WBD) (Almeida *et al*., 2017; Teixeira *et al*., 2015) caused by the fungal pathogen *Crinipellis perniciosa* has reduced cacao yields in many cultivation areas in South America including Ecuador and Brazil (Brown *et al*., 2005). In this context, a cacao osmotin‐like protein and various synthetic peptides (Falcao *et al*., 2016), and a phylloplanin (Freire *et al*., 2017) have been shown to have be involved in the response to WBD. Also with reference to pathogens, MALDI‐TOF MS methods have been applied recently for the rapid identification of *M. perniciosa*,* Phytophthora palmivora*,* P. capsici*,* P. citrophthora*,* P. heveae*,* Ceratocystis cacaofunesta*,* C. paradoxa* and *C. fimbriata* (dos Santos *et al*., 2017).

Another major disease problem in cacao is *Cacao swollen shoot virus* (CSSV) (Muller, 2016), which is transmitted largely by mealy bugs (Wetten *et al*., 2016). Although efforts have been made to eradicate the problem by removing infected trees, this has proved unsuccessful (Ameyaw *et al*., 2015) and it is now hoped that a greater understanding of the genetic variation in both the virus (Abrokwah *et al*., 2016; Chingandu *et al*., 2017a,2017b) and its vector (Herrbach *et al*., 2016), together with studies in more amenable model species (Friscina *et al*., 2017), will lead to progress in understanding this important disease and related badnaviruses (Andres *et al*., 2017; Bhat *et al*., 2016).

The second main breeding objective relates to physiological traits, as in addition to major pest and disease outbreaks, cacao cultivation is also affected by several other factors, which include altered short‐term climatic variation (e.g. El Niño), longer term global warming, high labour costs, depletion of soil fertility, poor plant productivity, lack of breeding strategies to develop and distribute improved varieties, and outdated farming practices (Zhang and Motilal, 2016). Specific breeding objectives reported in one recent study include dwarfism or semi‐dwarfism, which might enable smaller trees to be planted at higher density, and photosynthetic efficiency, an important determinant of yield (Pereira *et al*., 2017). As an alternative approach to breeding efforts to increase yields in the major production areas, some cocoa producers are now considering new regions that might allow an extension in the area under cultivation.

Importantly in terms of breeding strategies, cacao has a relatively longer juvenile period, namely 3–5 years. This makes selection of fruit‐specific traits in breeding programmes more time‐consuming and expensive, as the trees must be maintained for a longer period of at least three years to visually observe such characters in pods. Moreover, this crop is primarily outbreeding (i.e. SCA 6 and EET 75 cacao clones), and therefore, many populations are mostly heterozygous. This makes generation of inbred lines from crosses more labour‐intensive, and doubled haploid lines (Dunwell, 2010) are not easily generated. Moreover, the self‐incompatibility that exists in some of the cultivated cacao clones means that breeding populations are often highly heterogeneous with a wide range of yields (Royaert *et al*., 2011). However, it should also be noted that genetic variability does exist in cacao populations, and there are several self‐compatible cacao clones, such as CCN 51 and ICS 6 (Cervantes‐Martinez *et al*., 2006). Cacao trees also require a large area of land and high input of resources, including labour, for their maintenance under field conditions. These characteristics have made this crop less attractive as a model system, although like Arabidopsis, it has a relatively small genome.

Because of the recalcitrant (do not survive drying) nature of its seeds, the germplasm of this allogamous tree crop must be conserved in field genebanks as a living collection (Motilal *et al*., 2013) or by cryopreservation (Adu‐Gyamfi and Wetten, 2012; Adu‐Gyamfi *et al*., 2016). The largest collections of cacao germplasm are those at the International Cocoa Genebank, Trinidad (ICG, T) (https://sta.uwi.edu/cru/index.asp) (2400 accessions) and at the Centro Agronomico Tropical de Investigación y Enseñanza (CATIE) in Costa Rica (https://www.catie.ac.cr/en/) (1146 accessions), with another collection (c. 400 accessions), the International Cocoa Quarantine Centre, housed at the University of Reading, UK (http://www.icgd.reading.ac.uk/icqc/). However, maintaining genetic resources as living collections *in situ* or *ex situ* is practically difficult and is also an expensive process. A significant number of mislabelled accessions have been reported in these field genebanks (Motilal and Butler, 2003; Motilal *et al*., 2012). Thus, an efficient strategy to eliminate these mislabelled and/or duplicated accessions in large cacao germplasm collections is required for efficient and accurate management of genetic resources. In this context, DNA fingerprinting as a screening tool has been extensively used in rapid and accurate identification of cacao accessions. Restriction fragment length polymorphisms (RFLPs), random amplified polymorphic DNA (RAPD), amplified fragment length polymorphisms (AFLPs), microsatellites and single nucleotide polymorphisms (SNPs) are some of the molecular markers commonly used in cacao molecular studies (Kuhn *et al*., 2012; Lanaud *et al*., 1999; Laurent *et al*., 1994; Motilal and Butler, 2003; Santos *et al*., 2012a; Turnbull *et al*., 2004). Livingstone *et al*. (2012) report an optimized 5′‐nuclease (TaqMan)‐based SNP assay for efficient genotyping of cacao trees under field conditions. This simple, cost‐effective method would be a useful technique for cacao breeders.

Development of high‐density molecular‐linkage maps and characterization of molecular markers linked to major quantitative trait loci (QTL) have greatly accelerated breeding programmes in cacao by facilitating examination of particular fruit‐specific characters at a genotype level. The QTL mapping studies of this species have been performed using different mapping populations, that is F1 or F2 mapping populations (Lanaud *et al*., 2009; Motamayor *et al*., 2013; Royaert *et al*., 2011; Schnell *et al*., 2007) and association mapping or linkage disequilibrium mapping systems (Marcano *et al*., 2007; Stack *et al*., 2015). The first genomic map of cacao with a total of 193 loci covering 759 cM in 10 linkage groups was published by Lanaud *et al*. (1995). RFLP and RAPD markers were mainly used to construct this genetic map, which was subsequently used to produce high‐density molecular‐linkage maps in several subsequent studies. For example, Risterucci *et al*. (2000) published a high‐resolution molecular‐linkage map comprising 424 markers covering 885.4 cM over ten linkage groups; AFLP and simple sequence repeat (SSR) markers were employed to construct this high‐density map, which was considered as a good reference map for research activities in cacao (Clément *et al*., 2001). Development and mapping of codominant microsatellite markers to the cacao genome has accelerated genetic studies and breeding experiments. These PCR based codominant SSR markers are highly polymorphic, and easily transferrable between/across populations and/or laboratories; they can thus be used in MAS (Pugh *et al*., 2004). The high‐density linkage map described in this study has 465 markers with 268 SSR markers. A small number of such SSR markers have also been used in a study of genetic diversity in historical cacao plantations in Brazil (Santos *et al*., 2015) and germplasm assessments in Indonesia (Dinarti *et al*., 2015) and Cuba (Martínez *et al*., 2017). The collection of *T. cacao* expressed sequence tags (ESTs) generated from a range of organs, genotypes and environmental conditions is a valuable resource for discovery of important candidate genes and molecular markers for cacao genetic improvements (Argout *et al*., 2008). For instance, Fouet *et al*. (2011) discovered 174 EST‐based SSRs markers by screening a cacao EST data set and developed a high‐density linkage map with 582 codominant markers including 384 SSR markers. Most recently, da Silva *et al*. (2017) used a set of 20 EST‐SSRs to examine the evolutionary relationship of species within the *Theobroma* genus. Also, within the last few years, the use of SNP‐based codominant markers in genetics has increased significantly, due to advances in high‐throughput sequencing systems. A SNP‐based linkage map for cacao was initially developed by Allegre *et al*. (2012). This genetic linkage map contains a set of 1262 markers spanning in a length of 734 cM, and of these markers, 681 are EST‐based SNPs.

Recently, a large number of SNPs have been detected by aligning RNA sequence (RNAseq) data of 16 cacao cultivars to the assembled Matina 1–6 transcriptome (Livingstone *et al*., 2015). In this study, a saturated genetic linkage map with 2589 SNPs was constructed. More importantly, this study led to the development of an Illumina Infinium SNP array for cacao—Cacao6kSNP array that consisted of 6000 high‐quality SNPs. The newly developed array and the SNP data reported by Livingstone *et al*. (2015) provide a valuable genomic resource for cacao breeding. The latest genetic linkage map of cacao includes SNP data obtained from a large mapping population (459 trees) of a cross between WBD resistant, TSH 1,188 and WBD tolerant (moderately resistant to WBD) CCN (Royaert *et al*., 2016). It contains 3526 SNP markers and has a length of 852.8 cM. In addition to genetic linkage mapping studies, several recent studies have highlighted the importance of SNP‐based DNA fingerprinting in assessing cacao bean authentication (Fang *et al*., 2014), cacao variety development (Padi *et al*., 2015, 2017) and cacao genetic diversity (Cosme *et al*., 2016).

This rapid discovery of molecular markers also permits the efficient identification and study, in cacao, of the genetic basis of QTL for many agronomic traits such as bean traits and the number of ovules per ovary (Clement *et al*., 2003b), butter content and its hardness in cocoa beans (Araújo *et al*., 2009), diseases resistance for Ceratocystis wilt (Santos *et al*., 2012b), resistance for *Phytophthora* species (Akaza *et al*., 2016; Clement *et al*., 2003a; Efombagn *et al*., 2016; Flament *et al*., 2001; Lanaud *et al*., 2004; Legavre *et al*., 2015; Motilal *et al*., 2016; Risterucci *et al*., 2003), resistance for WBD (Brown *et al*., 2005; Faleiro *et al*., 2006; Motilal *et al*., 2016; Queiroz *et al*., 2003; Royaert *et al*., 2016), number of filled seeds (Motilal *et al*., 2016), yield (Clement *et al*., 2003a; Crouzillat *et al*., 2000) and self‐compatibility/incompatibility (Royaert *et al*., 2011; da Silva *et al*., 2016). Furthermore, meta‐analysis on QTL related to disease resistance in cacao has been performed by Lanaud *et al*. (2009). Such information would be of great use in MAS, which, to date, is employed in many crop breeding programs for development of improved cultivars including cacao. Recently, a semi‐automated genotyping platform for MAS known as amplicon sequencing (AmpSeq) has been successfully applied for grapevine breeding programme (Yang *et al*., 2016). This study showed the applicability of this strategy in heterozygous crop breeding by generation of AmpSeq markers for several traits with high breeding value including disease resistance in grapevine. It is possible that such a high‐throughput, cost‐effective, flexible and rapid breeding strategy could be implemented with some modifications to assist MAS in cacao in the future.

In addition to markers based on nuclear genomic DNA, several loci of chloroplast DNA (cpDNA) such as *matK*,* rbcL* and *trnH‐psbA* can be used as markers in DNA barcoding (Bieniek *et al*., 2015). Recently, sequence variation of the *trnH‐psbA* intergenic spacer has been analysed in 28 cacao accession obtained from different farms in southern Mexico (Gutiérrez‐López *et al*., 2016). It was found that the indels located in this region could be considered as potential markers for development of a DNA barcoding system in cacao. These markers are useful in identification of accessions in situations when other marker systems can only discriminate between accessions on the basis of a very small number of SSR markers (Gutiérrez‐López *et al*., 2016).

### Propagation methods and *in vitro* embryogenesis

Genetic improvement of cacao for improved traits has been hindered due to its narrow genetic base and long life cycle (Li *et al*., 1998). It is estimated that approximately 30% of world cacao production is lost due to pest and diseases, annually (Guiltinan *et al*., 2008). Therefore, an efficient propagation method for cacao is essential to accelerate breeding programmes and to avoid production shortages in the future. The cacao crop is grown with an approximate planting density of 1100 trees per hectare, and it has been estimated that with a replanting rate of 10%, there is an annual requirement for one billion units. This requirement is not being met at present, and some of the alternative propagation options (Laliberte and End, 2015) are considered below.

Cacao is normally propagated by means of seeds. Additionally, to maintain a genetically stable population, it is also propagated through a number of vegetative/asexual methods, of which a variety of grafting methods (Miguelez‐Sierra *et al*., 2017) are the most commonly practiced; these methods have been reviewed in detail by Sena Gomes *et al*. (2015). However, these propagation systems are not widely practiced in developing countries (Maximova *et al*., 2002). This could be due to the low rate of propagation and undesirable morphological features observed in some propagules, which often lack normal dimorphic nature and display bush‐like growth with a fibrous root system. Therefore, maintenance of such material is a more labour‐intensive process and requires skilled workers (Traore *et al*., 2003).

In cacao, *in vitro* embryogenesis or somatic embryogenesis (SE) is an alternative to traditional propagation methods and allows rapid clonal propagation of true‐to‐type plants with normal dimorphic architecture and taproot formation. Importantly, this system has shown to be an effective method in propagation of CSSV disease‐free plantlets (Quainoo *et al*., 2008). Moreover, a study conducted by López and co‐workers in 2010 found that *de novo* genetic mutations and epigenetic modifications do not accumulate with ageing of *in vitro* induced cacao calli (López *et al*., 2010b); this conclusion is also relevant to a recent study of long‐term SE (Quinga *et al*., 2017). Additionally, the SE system has been utilized in cacao germplasm conservation through cryopreservation (Adu‐Gyamfi and Wetten, 2012; Adu‐Gyamfi *et al*., 2016) and in genetic transformation (Maximova *et al*., 2002; da Silva *et al*., 2008). Induction of cacao somatic embryos has been observed from a range of its tissues, that is zygotic embryos (Pence *et al*., 1980), floral parts—petals and staminodes (Alemanno *et al*., 1996, 1997; Boutchouang *et al*., 2016; Li *et al*., 1998; Tan and Furtek, 2003), and nucellar tissues (Figueira and Janick, 1993). The SE method developed by Li *et al*. (1998) was applicable to many different cacao genotypes, and later, Maximova *et al*. (2002) improved this system to produce secondary somatic embryos from primary somatic embryos. Somatic embryo‐derived plants have been successfully grown under field conditions (Maximova and Guiltinan, 2015; Maximova *et al*., 2008). However, the efficiency of SE in this species is strongly influenced by genotype, particularly in respect of the conversion rate of mature somatic embryos into complete plants (Maximova *et al*., 2002). In addition, the type of explant used and its position, that is flower bud position (Boutchouang *et al*., 2016; Traore and Guiltinan, 2006), tissue culture media composition (Traore and Guiltinan, 2006) and phenological parameters such as the periodicity of new leaf development (Issali *et al*., 2008) are also have an impact on SE efficiency in cacao. Several researchers have focused on optimizing *in vitro* culture media composition to improve somatic embryo differentiation in this species (Minyaka *et al*., 2008; Niemenak *et al*., 2008, 2012; Traore and Guiltinan, 2006). Furthermore, establishment of a temporary immersion bioreactor system for mass production of cacao somatic embryos was a breakthrough process in cacao biotechnology research (Niemenak *et al*., 2008). Consequently, studies were designed to further optimize somatic embryo induction in liquid suspension cultures (Niemenak *et al*., 2012). More recently, an alternative method to induce cacao primary SE at a high efficiency has been achieved by supplementing DKW medium with different ratios of kinetin to 2,4‐dichlorophenoxyacetic acid (1.0: 3.9 callus induction medium; 1.0: 7.8 secondary callus growth medium) (Ajijah *et al*., 2016). Furthermore, this methodology yielded a 65% plantlet conversion rate and a relatively low percentage of somaclonal variation (López *et al*., 2010a). Other beneficial modifications to SE media have been reported recently by Kouassi *et al*. (2017) and Modeste *et al*. (2017).

Further details and modified SE protocols developed by the various commercial chocolate companies are also given in the various patent documents mentioned in the section below. Despite continuous progress, the overall low efficiency and reproducibility of the methods developed as well as the genotype dependent nature of the many steps involved in the SE process still present a significant challenge for mass propagation of many elite cacao genotypes at the commercial scale required for many parts of the cacao growing regions (da Silva *et al*., 2008). In an ambitious scheme, Nestlé's Cocoa Plan aims to produce and distribute at least 12 million plants of elite varieties that are disease free, high yielding and high quality in terms of beans and taste by the year 2022 (Fair Labor Association, 2012; Guillou *et al*., 2014). They aim to improve farmers’ income and living conditions and avoid deforestation through sustainable production of cocoa, technology transfer and distribution of quality planting material for propagation in Côte d'Ivoire.

Understanding the molecular mechanism of SE would allow us to improve this process in economically important crops, including cacao, and thereby provide an efficient system to speed‐up commercial plant production of many crops in the coming years. Recent advances in high‐throughput sequencing systems and ‘omics’ resources have facilitated generation of high‐resolution transcriptome data for plant embryogenesis, both *in vivo* and *in vitro* and thereby, to provide novel insights into the molecular basis of embryogenesis (Wickramasuriya and Dunwell, 2015; Xu *et al*., 2012). In a relevant recent example in cacao, gene expression profiles of zygotic embryogenesis (ZE) and SE have been generated using whole‐genome microarray (Maximova *et al*., 2014). This study reported that a large number of genes including those encoding for transcription factors, genes related to flavonoid and lipid biosynthesis were differentially expressed between the two embryo developmental processes. Such results thus provide an insight into cacao SE at a molecular level, and the information provided could be used to develop and characterize novel molecular markers for SE. In addition, proteome profiles of cacao SE and their equivalent ZE at various developmental stages have been generated and analysed through 2D PAGE and nano‐LC‐MC (Niemenak *et al*., 2015; Noah *et al*., 2013). Availability of the genome sequence together with such recent proteomic and transcriptomic information for cacao embryogenesis provides a good starting point for functional studies of many genes and their encoded proteins essential for embryo development in this species.

In addition, several key regulators of plant embryogenesis have also been isolated and characterized from cacao. Of these, the members of the Leafy cotyledon (LEC) gene family—*LEC1*,* LEC2* and *FUSCA3* (*FUS3*)—serve as master regulators of embryo development, and they have been well characterized in Arabidopsis (Lotan *et al*., 1998; Stone *et al*., 2001). Recently, a functional ortholog of Arabidopsis *LEC2* has been isolated and characterized in *T. cacao* (*TcLEC2*) by Zhang *et al*. (2014). This gene was found to be expressed at a significant level in endosperm and cotyledons but not in flower and leaf tissues (Zhang *et al*., 2014). Furthermore, a 20‐fold higher level of *TcLEC2* transcript accumulation was observed in embryogenic calli than in nonembryogenic calli (Zhang *et al*., 2014). Moreover, overexpression of *TcLEC2* has led to increased expression of several seed‐specific genes in leaves of cacao, that is *TcAGL15* (>129‐fold), *TcABI3* (>9‐fold) and *WRINKLED1* (*WRI1*) (>10‐fold). This also increased the embryogenic competency of cotyledon explants and regeneration capacity of somatic embryos, supporting the fact that, as in many other plants, *TcLEC2* is a key regulator of cacao embryogenesis. Furthermore, a functional homologue of the *LEC1*‐*like* gene has also been reported from the cacao genome (Alemanno *et al*., 2008); increased expression of this gene has been detected in early stages of cacao zygotic and somatic embryogenesis.

Another well‐studied regulator of embryogenesis is the AP2/ERF family member BABY BOOM (BBM), which was first identified from *Brassica napus* microspore‐derived *in vitro* embryos (Boutilier *et al*., 2002). Being significantly expressed in developing embryos and seeds, *BBM* is considered as one of the key marker genes in embryogenesis (Boutilier *et al*., 2002; Ikeda *et al*., 2006; Karami *et al*., 2009). A functional gene with a high degree of similarity to *BBM* in *A. thaliana* has been isolated and characterized from cacao (*TcBBM*) (Florez *et al*., 2015). *TcBBM* expression has been detected throughout embryogenesis in cacao; a higher level of expression has been detected in SE than in ZE. In a manner similar to that in species including Arabidopsis (Boutilier *et al*., 2002) and cereals (Lowe *et al*., 2016), overexpression of *TcBBM* in cacao has been found spontaneously to induce somatic embryos in hormone‐free media (Florez *et al*., 2015); thus, *TcBBM* could be used to enhance the efficiency of SE in cacao. The most recent advance in this area is the report of an inducible SE system by exploiting a dexamethasone activatable embryogenic transcription factor to promote somatic embryo formation from juvenile leaves (Shires *et al*., 2017).

Similarly, kinases play an important role in plant embryogenesis. For example, *somatic embryogenesis receptor kinases* (*SERK*s) are a subgroup of protein kinase genes that are expressed in early stages of somatic and zygotic embryo development. These genes were initially isolated from *in vitro* embryogenic cultures of carrot by Schmidt *et al*. (1997) and have subsequently *SERK*s been identified and characterized in many species, that is *A. thaliana* (Hecht *et al*., 2001), *Solanum tuberosum* (Sharma *et al*., 2008), *Cocos nucifera* (Perez‐Nunez *et al*., 2009)*, Zea mays* (Baudino *et al*., 2001), *Momordica charantia* (Talapatra *et al*., 2014) and cacao (de Oliveira Santos *et al*., 2005). This latter gene (*TcSERK*) was highly expressed in embryogenic calli, and also in mature zygotic and somatic embryos at a moderate level, suggesting that the functional copy of *SERK* found in cacao plays a key role during the process of embryo development.

## Genetic transformation

Completion of whole‐genome sequencing for many economically important crops has significantly contributed to their respective genetic improvement. In addition, identification and functional characterization of novel genes and transfer of genes that regulate agronomically valuable traits such as disease resistance have been achieved in many crops. The earliest attempt of cacao transformation was recorded in 1994 by Sain *et al*. (1994) using the Agrobacterium‐mediated gene transfer method. Although transformed callus cells derived from leaf tissues were obtained, no plant regeneration was recorded from those transformed cells. This was due to the lack of an efficient protocol to recover plants from cacao leaf tissue‐derived calli at that time (Sain *et al*., 1994).

Subsequently, a more efficient method for stable genetic transformation and recovery of transformed plants from transformed cacao cells was established by Maximova *et al*. (2003). This study employed SE as a regeneration system together with *Agrobacterium tumefaciens* cocultivation to obtain transgenic plants. This system has been successfully used to produce transgenic cacao plants overexpressing cacao class I *Chitinase* gene (*TcChil1*) (Maximova *et al*., 2006). These transgenic plants showed enhanced fungal pathogen resistance against *Colletotrichum gloeosporioides*. Although this transformation system was proven to be reproducible (Maximova *et al*., 2003, 2006), the reliable production of a large number of transgenic embryos remains a challenge. Subsequently, several studies have been conducted in an attempt to improve the efficiency of the transformation method described in Maximova *et al*. (2003) (Silva *et al*., 2009). Most recently, an optimized method for transient transformation of cacao for several genotypes through Agrobacterium infiltration has been published by Fister *et al*. (2016b). As a tool, this will allow more efficient *in vivo* functional analysis of cacao genes, subcellular localization of proteins and promoter analysis. It is also possible that the use of rapidly flowering transgenic lines, as used in other perennial species (Callahan *et al*., 2016), may be applicable to cacao. Most recently, an inducible SE system in cacao has been developed by use of a transgenic plant expressing the LEC2 embryogenic transcription factor (Shires *et al*., 2017).

Although much effort has been devoted to improve cacao varieties through genetic transformation, genetically modified (GM) cacao material has not been released commercially so far, and the studies have been limited to laboratories and greenhouses. Although genetic transformation serves as a valuable tool in crop improvements, the future of GM cacao is not clear as the consumer acceptance of food from genetically modified organisms is still a controversial issue in some countries (Dunwell, 2014; Guiltinan *et al*., 2008).

It is now possible to effect gene‐specific mutagenesis by genome editing, (e.g. CRISPR/Cas9) for functional characterization of genes; this also serves as a promising approach for genetic manipulation of disease resistance, and other genes in cacao. Whether or not such methods will be considered as a form of GM is still uncertain, at least in Europe.

### Resources available for cacao functional studies

Open‐source bioinformatic tools and web databases have greatly contributed to the rapid developments in omics‐based researches and thereby crop improvements. A list of tools/databases freely accessible for cacao researchers and breeders is summarized in Table 1. The latest genome sequence of cacao is available on the Cacao Genome Database (CGD, http://www.cacaogenomedb.org/), which was developed in collaboration with MARS, USDA/ARS, IBM, Clemson University Genomics Institute, PIPRA, HudsonAlpha Institute for Biotechnology, National Center for Genome Resources, Indiana University and Washington State University—Main Lab Bioinformatics. In addition, tools such as BLAST and GBrowse can be accessed through this web database.

**Table 1:** A list of web based resources available for cacao

Resource	URL
CacaoNet—Global Network for Cacao Genetic Resources	https://sites.google.com/a/cgxchange.org/cacaonet/home/partners-of-cacaonet
International Cocoa Germplasm Database (ICGD)	http://www.icgd.reading.ac.uk/index.php
Cacao Genome Database	http://www.cacaogenomedb.org/
Cocoa Genome Hub	http://cocoa-genome-hub.southgreen.fr/
CocoaGenDB	http://cocoagendb.cirad.fr/
CEMID—Cocoa EST Marker Information Database	http://riju.byethost31.com/cocoa/?ckattempt=1
Ensembl Plants	http://plants.ensembl.org/Theobroma_cacao/Info/Index
Dicots PLAZA 3.0	http://bioinformatics.psb.ugent.be/plaza/versions/plaza_v3_dicots/organism/view/Theobroma+cacao
Phytozome v11.0	https://phytozome.jgi.doe.gov/pz/portal.html#!info?alias=Org_Tcacao
GenBank NCBI	http://www.ncbi.nlm.nih.gov/genome/?term=cocoa
Witches’ Broom Disease Transcriptome Atlas	http://bioinfo08.ibi.unicamp.br/wbdatlas/

### Survey of patents relating to cacao

Often, very useful information about advances in scientific research can be obtained from a study of patent databases, as information is often published here before appearing in more usual scientific publications (Dunwell, 2012). In addition to academic institutes, private sector industries play a key role in cacao research and development. Hence, a patent search analysis was performed using the Lens patent database (https://www.lens.org/lens/) to provide an overview of public and private sector involvement, and application of biotechnology techniques in cacao research; structured search was carried out on granted patents using the terms ‘Theobroma ‘ or ‘cacao’ or ‘cocoa’ in the abstract or claims. A total of 2732 granted patents were recorded prior to 13 July 2017. Thus, because it is not feasible to summarize all these granted patents in this review only a selected summary is provided here (Tables 2). In brief, granted patents related to plant breeding and biotechnology applications were filtered based on the International Patent Classification (IPC) codes; a total of 10 granted patents with following IPC codes were identified and listed in Table 2: A01G 17/00, A01H 4/00, A01H 5/08, C12N 5/00, C12N 5/02, C12N 5/04, C12N 15/82, C12N 15/84 and C12N 15/87 (definition of these IPC codes can be obtained through the World Intellectual Property Organization (WIPO) page http://www.wipo.int/classifications/ipc/en/). We identified several granted patents relating to the production of cacao somatic embryos through optimized tissue culture techniques. Not surprisingly, several studies are funded either fully or partially by the leading chocolate manufacturers. Notable publications of particular relevance to this review include those describing methods for SE with granted patents from Hershey Foods (US 5312801) in 1994, Penn State Research Foundation (US6150587) in 2000, Nestle S.A. (US 8921087) in 2014, and most recently a granted patent from Mars Inc. (AU 2014/353082 B2) in April 2017. This patent on the production of cacao plants claims micropropagation via direct SE and is available at https://www.lens.org/images/patent/AU/2014353082/B2/20170420/AU_2014_353082_B2.pdf. This method uses explants such as staminodes and petal base tissues for induction of primary embryos in a medium supplemented with 6‐benzylaminopurine (BAP). Subsequently, the epicotyl segments removed from primary embryos are placed in an induction medium containing BAP to induce direct secondary embryos, followed by further embryo development in a medium containing gibberellic acid, if needed. All the cultures are maintained in the light (photoperiod: 16 : 8 (light: dark) at a temperature of 23–29 °C for a sufficient period of time to obtain embryos. Although several attempts have been made in the past to develop a direct SE system for cacao micropropagation, there has been only limited success (Pence *et al*., 1980). Therefore, the detailed information provided in this patent related to a direct SE method may provide additional valuable information for cacao researchers working on this subject. However, there is no patent information yet on the use of novel breeding techniques such as Zinc Finger Nuclease (ZFN) or CRISPR/Cas9 technology, cisgenesis and RNA‐dependent DNA methylation (RdDM) in cacao plants.

**Table 2:** A list of granted patents, in chronological order, on *T. cacao* biotechnology, prior to July 2017

Publication Number	Publication Year	Title	Applicant(s)
US 4301619 A	1981	Plant tissue produced by non‐agricultural proliferation of Cacao embryos	Purdue Research Foundation [owner]
US 4291498 A	1981	Method for production of mature asexual Cacao embryos, and product thereof	Purdue Research Foundation
US 4306022 A	1981	Cocoa bean cell culture	Cornell Research Foundation Inc
US 4545147 A	1985	Asexual embryogenesis of callus from *Theobroma Cacao* L.	Purdue Research Foundation [owner]
US 5312801 A	1994	Somatic embryogenesis and plant regeneration of Cacao	DNA Plant Technology Corporation
			Hershey Foods Corporation [owners]
US 6150587 A	2000	Method and tissue culture media for inducing somatic embryogenesis, *Agrobacterium*‐mediated transformation and efficient regeneration of Cacao plants	Penn State Research Foundation [owner]
US 8921087 B2	2014	Cocoa somatic embryogenesis	Florin Bruno Jean‐Marie
			Masseret Bernard
			Vachet Caroline Denise Monique
			Nestec SA[owner]
US 8969655 B1	2015	Modulation of flavonoid content in Cacao plants	Daniel Preston
			Randall B. Murphy
			Cacao Biotechnologies LLC [owner]
US 9428759 B2	2016	Methods for increasing the production of phenolic compounds from *Theobroma Cacao*	Rengifo Raul Cuero
			Casa Luker SA [owner; International Park of Creativity]
AU 2014/353082 B2	2017	Production of plants using somatic embryogenesis	Mars Inc [owner]

## Conclusion

There is a great demand for high‐quality cocoa beans. Thus, to ensure long‐term sustainability of cocoa production, future research should focus on the development of improved cacao varieties that can both tolerate changing climates, but also meet the stringent quality criteria demanded by the chocolate industry. Implementation of modern molecular tools in cacao biotechnology research will undoubtedly be an integral part of this process.

## Conflict of Interest

The authors declare no conflict of interest.
